# Fracture Incidence With 6–9 Years of Daily School Physical Activity: Lower Relative Fracture Risk at the End of the Intervention But Not After

**DOI:** 10.1111/sms.70319

**Published:** 2026-06-11

**Authors:** Jakob Rempe, Magnus Dencker, Otto Nilsson Wimar, Lars Jehpsson, Björn E. Rosengren, Magnus K. Karlsson

**Affiliations:** ^1^ Department of Orthopedics Helsingborg Hospital, Lund University Helsingborg Sweden; ^2^ Orthopedics, Faculty of Medicine, Department of Orthopedics and Clinical Sciences Lund University, Skane University Hospital Malmo Sweden; ^3^ Department of Physiology, Clinical Sciences Lund University, Skane University Hospital Malmo Sweden

**Keywords:** children, fracture, intervention, physical activity, prevention, school

## Abstract

Fractures pose a significant burden on society, making prevention strategies important. One approach is to increase physical activity (PA), which may enhance bone mass and muscle strength, thereby lowering fracture risk. A total of 3476 children who began 1st grade (mean age 7 years) between 1998 and 2012 in four Swedish schools were included. The intervention group was 1328 children from one school who received daily school PA (200 min/week, 5 lessons/week) for a mean of 6.9 (SD 1.3) years. Controls were 2148 age‐matched children in three schools who received the Swedish standard of PA (60 min/week, 1–2 lessons/week). Fractures were identified in the regional radiographic archives. Participants were followed until death or study end (2024‐08‐31). Participants who migrated from the region were excluded during periods away. This resulted in a mean follow‐up period of 8.2 (SD 4.3) years after the intervention. We used incidence rate ratios (IRRs) to express differences between groups. We identified 1278 fractures in 1225 events. IRR (intervention group compared to controls) was in lower primary (ages 7–9) 1.22 (95% confidence interval (CI) 0.93–1.60), in upper primary (ages 10–12) 1.14 (95% CI 0.91–1.43), and in lower secondary (ages 13–15) 0.78 (95% CI 0.61–0.99). After the intervention (ages 16–30), IRR was 1.04 (95% CI 0.85–1.27). The study indicates that daily school PA initiated at age 7 is associated with a lower relative fracture risk at the end of the intervention (ages 13–15) but not after termination of the intervention. Our data indicate that daily school PA may possibly be one method to decrease fracture risk during peak fracture incidence at growth.

**Abbreviations**
CI: Confidence interval
IR: Incidence Rate
IRR: Incidence Rate Ratio
PA: Physical Activity
POP: Pediatric Osteoporosis Prevention Study
SD: Standard Deviation

## 1 Introduction

Fractures are common in both children and the elderly, leading to individual suffering and high costs for society [1]. Before age 17, almost one third of children sustain a fracture, reaching a peak around puberty [2]. The incidence of pediatric fractures has increased in Malmo, Sweden, from 1950 to 1979 by around 1.5% per year [3], but has been stable thereafter [4]. However, bone mass in children has been reported to be lower than it was four decades ago [5], possibly influenced by factors such as increased screen time [6, 7] and decreased physical activity (PA) [8, 9]. As low bone mass is associated with higher fracture risk in both children [10] and adults [11], the future fracture incidence may therefore increase.

The number of fractures has also increased among adults in recent decades [12]. This could partly be explained by demographic shifts in the population, as longer life expectancy leads to a larger elderly population [12]. Although age‐ and gender‐specific hip fracture incidence decreased during recent decades [13], earlier projections suggested that the demographic shift would lead to a doubling of the number of hip fractures between 2002 and 2050 [14]. However, a more recent Swedish projection published in 2024 predicted that a continued decline in hip fracture incidence could result in 30% fewer hip fractures among the Swedish population by 2050 [15].

There are many risk factors for fractures [16, 17, 18, 19]. Some, such as age, sex, and heredity, are unmodifiable [16] and could be used to target high‐risk individuals. Modifiable risk factors, such as low physical activity (PA), low bone mass, smoking, use of glucocorticoids, excessive alcohol consumption, low calcium intake, low vitamin D levels, poor balance, and impaired vision [17, 18, 19], may be addressed through interventions. With fractures being a major health problem and projections indicating higher numbers in the future, it is important to identify effective, inexpensive prevention strategies that could be implemented on a population‐based level without adverse effects. Increased PA may be such an intervention, the hypothesis supported by previous studies from the Pediatric Osteoporosis Prevention (POP) study, showing that daily school PA from before to after puberty is associated with beneficial gain in bone mass and muscle strength [20] and reduced risk over time for fractures [21]. There are, however, indications that initiation of a daily school PA program might be associated with a temporary increase in relative fracture risk [21]. Furthermore, fracture risk after termination of the intervention is unknown. However, as the intervention has been reported to be associated with residual benefits in bone mass a mean of 7 years after termination of the program [22], it seems possible that the benefits may be associated with a lower relative fracture incidence also after the intervention has terminated. The finding of lower relative fracture risk in former athletes compared to controls [23] supports the view that PA during growth may have long‐term beneficial effects on fracture risk.

Based on the publications discussed above, we hypothesized that an intervention with 6–9 years of daily school PA during compulsory school years is associated with lower relative fracture risk both in lower secondary school (grades 7–9) and after termination of the intervention. We asked the following research questions: is an intervention with daily school PA (compared to standard school PA 1–2 times per week) associated with (i) a temporary higher fracture incidence, (ii) a lower fracture incidence at the end of the intervention, and (iii) a lower fracture incidence after the intervention is terminated?

## 2 Materials and Methods

### 2.1 The Pediatric Osteoporosis Prevention (POP) Study

The POP study is a population‐based controlled prospective PA intervention study investigating the effect of daily school PA on various health outcomes, with the study protocol detailed in previous publications [24, 25]. In summary, four neighboring elementary schools in a middle‐class area of the southwestern part of Malmo in the region Skane in southern Sweden, with similar socioeconomic and ethnic demographics within their catchment areas, were included. All schools were government‐funded, and students were assigned to their respective schools based on their residential address [24, 25]. Before the study began, all four schools practiced the Swedish standard curriculum of school physical activity (PA). Four schools were invited, and the first to accept became the intervention school, and the three remaining were control schools. The present study focuses on possible associations between the PA intervention and relative fracture risk compared to controls.

### 2.2 The Intervention

The intervention, an increase of the Swedish curricular standard of 60 min of school PA per week to 200 min per week (40 min per school day), started the first week in grade one (mean age 7), except for those who started school in 1998, where the intervention began the first week in grade two (mean age 8). The intervention continued the nine compulsory school years in Sweden (through grade 9; mean age 15) or until the fall of 2010. From that time point, the school reduced the PA curriculum in lower secondary school (grades 7–9) to three 40 min sessions/week. Due to this change in the school PA curriculum, the intervention group included 291 children with 9 years of daily PA, 124 with 8 years, 68 with 7 years, and 845 with 6 years (mean intervention 6.9 (SD 1.3) years). The control schools continued with the Swedish standard of PA (60 min of PA in 1–2 lessons per week) throughout the study period.

The regular teachers supervised the PA classes in all schools without additional resources. The classes included standard curricular activities such as running, jumping, ball games, dancing, swimming, and climbing. No specific activities designed to be osteogenic were added. All students had to participate as school PA is a compulsory subject in Sweden. During weekends and school vacations, there was no PA provided by the schools.

### 2.3 Study Cohort

At study start, 3494 children who began 1st grade in any of the four schools between 1998 and 2012 were included. Children who, during the study period, changed from the intervention school to the control schools, or vice versa, were excluded (*n* = 18). Of the remaining 3476 children, 1328 (46% girls and 54% boys) were in the intervention and 2148 (49% girls and 51% boys) in the control group. Information on migration was acquired from the Swedish National Population Registry and was used to exclude periods when a participant lived outside the region Skane (and thus was unable to contribute to the outcome fractures, as these were registered in the region Skane radiographic archives). From the Swedish Tax Agency, information on mortality was obtained, and study participants who died during the study period were included until the date of death (*n* = 3 in the intervention and *n* = 1 in the control group).

### 2.4 Fracture Registration

For each individual, we retrospectively searched the radiographic archives for fractures identified by radiologists in the general health care system. We included fractures from the first day in grade one to the study end date (2024‐08‐31). Children who started school in 1998 were thereby followed for 26 years (until age 33), and children who started school in 2012 for 12 years (until age 19). This resulted in fewer participants each year after the age of 19. Until 2001‐03‐11, fractures were identified in the analog radiological archive at Skane University Hospital (SUS) in Malmo, Sweden, the only emergency hospital in the city, and a hospital that saves all radiographs [26, 27]. The radiographic department at SUS changed its system on 2001‐03‐11 to digital radiographs. The new digital archive includes all radiographs taken within the general health care system in the region Skane, Sweden. Also, this archive has been described in detail and used in previous epidemiological reports over the last two decades [26]. If a patient had been diagnosed with a fracture at another hospital, the fracture was registered at follow‐up visits at the regional hospital through radiographic follow‐up. This method to objectively capture and classify fractures, detailed in previous studies, has been used for decades at our research center [26, 27]. Since we used the radiographic archives to register fractures by personal identity number, we could screen all the included participants.

All diagnosed fractures were retrospectively classified by one of the authors, a senior consultant in Orthopedics (MK), following the ICD‐10 classification. Referrals and reports were used to register trauma mechanisms according to the Landin classification [3]. Sporting injuries and falls at the same level were classified as low‐energy; traffic accidents not involving motor vehicles, falls on stairs, and falls from above the same level up to three meters were classified as moderate‐energy; and traffic accidents involving motor vehicles or falls from heights greater than three meters were classified as high‐energy [3].

Fractures were categorized by school grade according to the Swedish school year. In general, school starts in Sweden the year a child turns 7. The cutoff date for each school year was set to August 31. A fracture in grade 1 was thus defined as a fracture occurring between September 1 of the year the participant turned 7 until August 31 of the following year; a fracture in grade 2 was defined analogously for the year the participant turned 8, and so on. We followed the Swedish classifications of school levels, so that grades 1–3 were classified as lower primary school (ages 7–9), grades 4–6 as upper primary school (ages 10–12), and grades 7–9 as lower secondary school (ages 13–15). Older individuals, that is, aged 16 and above, were classified as post‐intervention. As few individuals were followed beyond age 30 (only those with school start from 1998 to 2000), we report fracture data only to age 30.

### 2.5 Statistics

We used R version 4.5.0 for statistical analyses. Data are presented as absolute numbers or means with 95% confidence intervals (95% CI). We calculated incidence rates (IRs) per 1000 person‐years with 95% CI, assuming a Poisson distribution for the number of fracture events in each age group [28], and incidence rate ratios (IRRs) by dividing the incidence in the intervention group by that in the control group. The 95% CIs for the IRRs were estimated using the method proposed by Rothman and Greenland [28]. We finally visualized the annual sex‐specific fracture incidence rates using three‐year moving averages.

### 2.6 Ethical Considerations

The study was approved by the Ethics Committee of Lund University (D.nr. LU 453–98 and D.nr. LU 2015/118) and the Swedish Ethical Review Authority (D.nr. 2019–02965). The study was conducted in accordance with the Declaration of Helsinki 2000. The clinical trial is registered at ClinicalTrials.gov (NCT00633828).

## 3 Results

**FIGURE 1:**
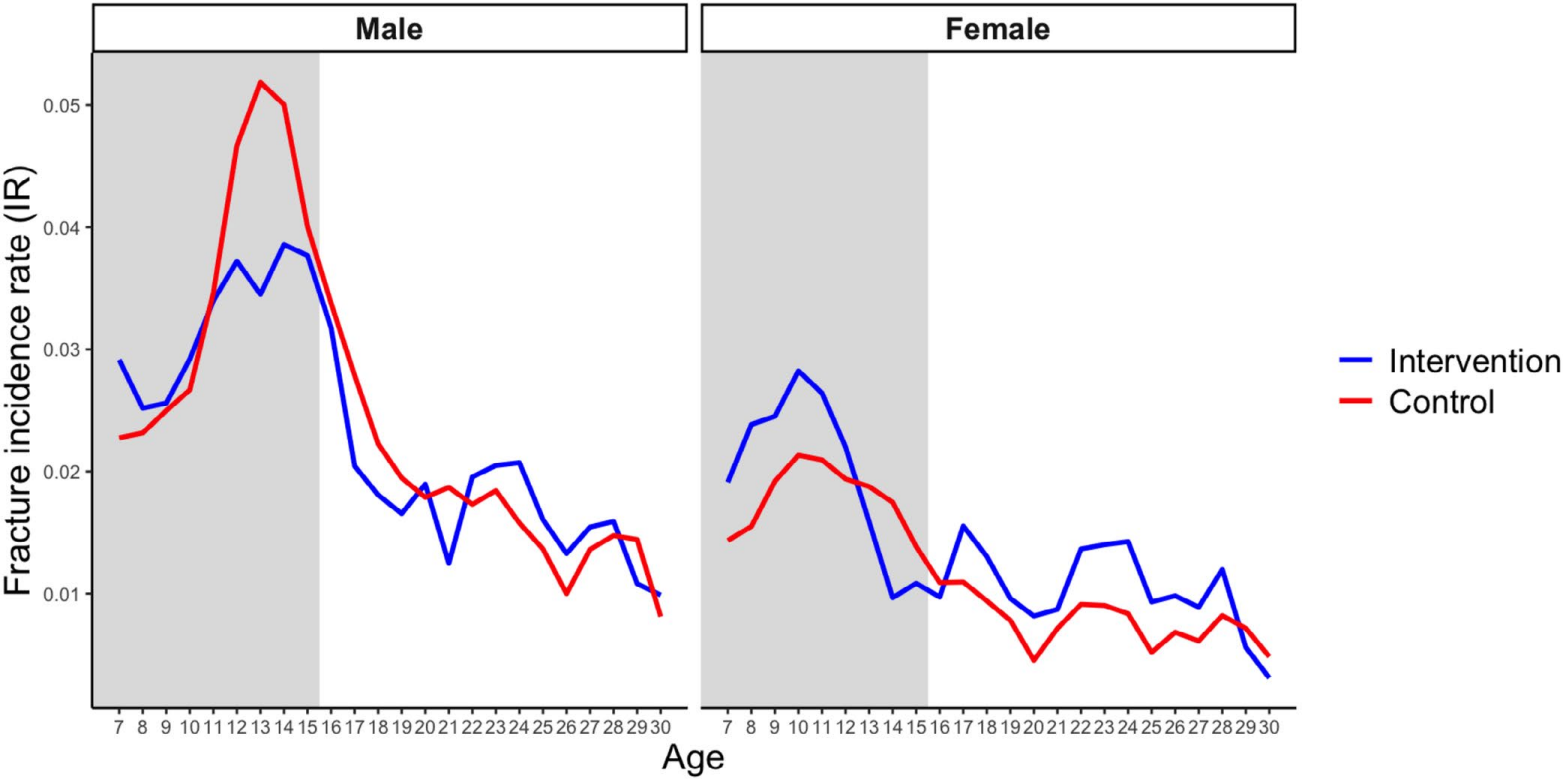
Descriptive visualization of sex‐specific fracture incidence rates (IR) per 1000 person‐years using three‐year moving averages. The gray area illustrates the intervention period (grades 1–9, mean 6.9 years of daily school‐based physical activity) and the white area the post‐intervention period.

During the 58 092 evaluated person‐years, we found 1278 fractures, sustained in 1225 specific fracture events. Fracture types and trauma mechanisms during and after the intervention period are presented in Table 1. The most common fracture location during the intervention period (ages 7–15) was the forearm, and in the post‐intervention period (ages 16–30) the hand. Most fractures, both during and after the intervention period, were the result of low‐energy trauma (Table 1). The sex‐specific moving averages of fracture incidence in the intervention and the control groups are shown in Figure 1.

**TABLE 1:** Types of fractures and trauma mechanisms associated with the total 1278 fractures, during the intervention period (grades 1–9, mean 6.9 years of daily school‐based physical activity; gray area), and the post‐intervention period (post‐grade 9; white area).

	Intervention period		Post‐intervention	
	Grades 1–9 (ages 7–15)		Post‐grade 9 (ages 16–30)	
	Intervention group	Control group	Former intervention group	Control group
Fracture type				
Forearm	141 (43.9%)	201 (38.8%)	27 (17.0%)	40 (14.3%)
Hand	77 (24.0%)	149 (28.8%)	53 (33.3%)	101 (36.1%)
Shoulder and upper arm	46 (14.3%)	57 (11.0%)	9 (5.7%)	22 (7.9%)
Lower leg and ankle	22 (6.9%)	48 (9.3%)	24 (15.1%)	33 (11.8%)
Foot	29 (9.0%)	40 (7.7%)	22 (13.8%)	48 (17.1%)
Femur	1 (0.3%)	8 (1.5%)	4 (2.5%)	10 (3.6%)
Spine and pelvis	0 (0%)	8 (1.5%)	2 (1.3%)	5 (1.8%)
Skull and facial bone	3 (0.9%)	1 (0.2%)	12 (7.5%)	16 (5.7%)
Other fracture	2 (0.6%)	6 (1.2%)	6 (3.7%)	5 (1.8%)
Trauma energy				
Low‐energy	195 (60.7%)	325 (62.7%)	107 (67.3%)	174 (62.1%)
Moderate‐energy	96 (29.9%)	128 (24.7%)	27 (17.0%)	55 (19.6%)
High‐energy	9 (2.8%)	21 (4.1%)	13 (8.2%)	25 (8.9%)
Undetermined	21 (6.5%)	44 (8.5%)	12 (7.5%)	26 (9.3%)
Total fractures	321	518	159	280

*Note:* Data are presented as absolute numbers (*n*) with proportions (%) of all fractures within each group and period provided within brackets.

**FIGURE 2:**
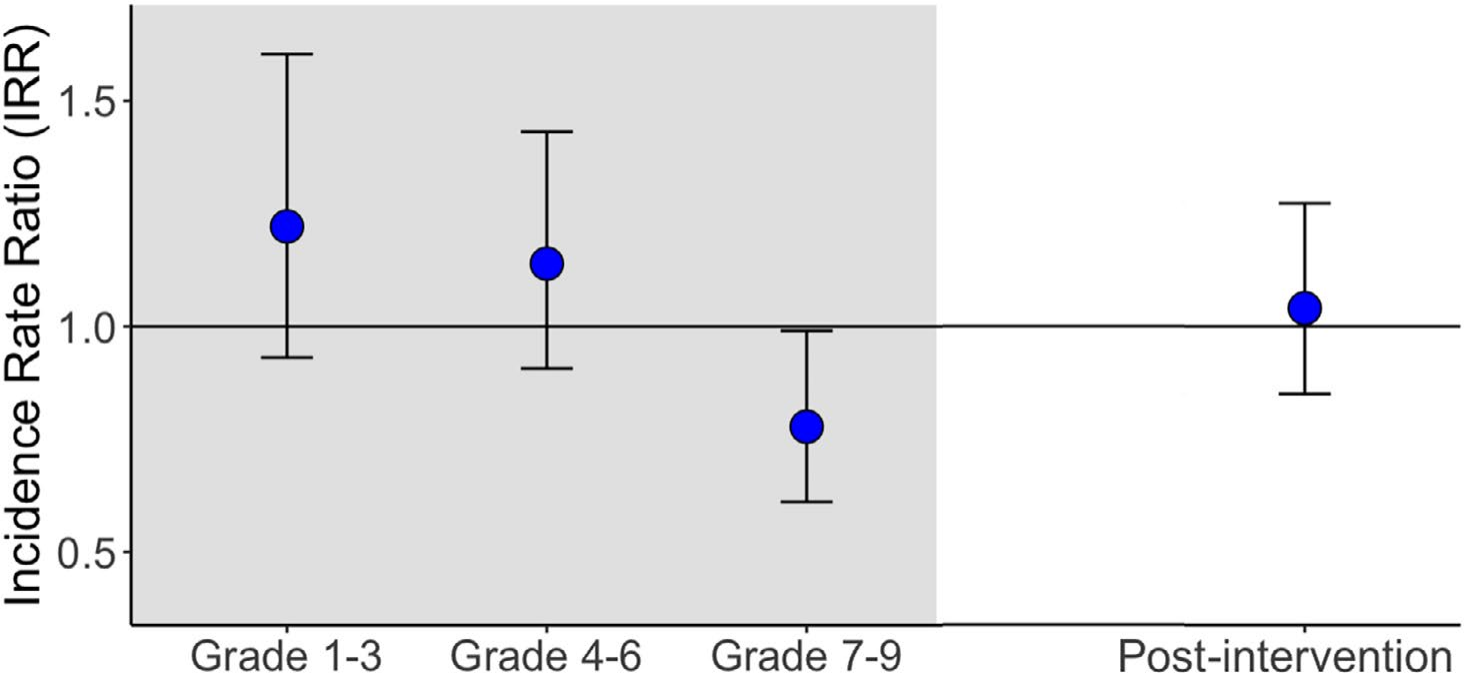
Incidence rate ratios for fracture events (IRRs) when comparing incidence rate (IR) in the intervention group with the control group during the intervention period (grades 1–9; ages 7–15, mean 6.9 years of daily school‐based physical activity), and the post‐intervention period (ages 16–30). The blue points represent the point estimates of IRRs for fracture events, and the bars the 95% confidence intervals (95% CI). The gray area illustrates the intervention period, and the white area the post‐intervention period. We regarded the result as statistically significant if the 95% CI for IRR did not include 1.0.

Fracture incidence rates and IRRs are presented in Table 2 and Figure 2. During the intervention, IRR was in lower primary school (ages 7–9) 1.22 (95% CI 0.93–1.60), in upper primary school (ages 10–12) 1.14 (95% CI 0.91–1.43), and in lower secondary school (ages 13–15) 0.78 (95% CI 0.61–0.99). In the post‐intervention period (ages 16–30), IRR was 1.04 (95% CI 0.85–1.27) (Table 2). Sex‐specific incidence rate ratios (IRR) are presented in Appendix 1.

**TABLE 2:** Fracture events (*n* = 1225) presented separately in the intervention group (mean 6.9 years of daily school‐based physical activity), and the control group, during the intervention period (grades 1–9; gray area), and the post‐intervention period (post‐grade 9; white area).

	Intervention period			Post‐intervention
	Grades 1–3 (ages 7–9)	Grades 4–6 (ages 10–12)	Grades 7–9 (ages 13–15)	Post‐grade 9 (ages 16–30)
Intervention group				
Individuals (*n*)	1328	1302	1273	1271
Person‐years (years)	3722	3835	3775	10 023
Fracture events (n)	91	125	97	148
Fracture events/1000 person‐years (IR) (mean (95% CI))	24.5 (19.7, 30.0)	32.6 (27.1, 38.8)	25.7 (20.8, 31.4)	14.8 (12.5, 17.3)
Control group				
Individuals (n)	2148	2115	2083	2087
Person‐years (years)	6043	6254	6202	18 238
Fracture events (n)	121	179	205	259
Fracture events/1000 person‐years (IR) (mean (95% CI))	20.0 (16.6, 23.9)	28.6 (24.6, 33.1)	33.1 (28.7, 37.9)	14.2 (12.5, 16.0)
Incidence rate ratio fracture events (IRR) (mean (95% CI))	1.22 (0.93, 1.60)	1.14 (0.91, 1.43)	**0.78 (0.61, 0.99)**	1.04 (0.85, 1.27)

*Note:* Data are presented as number (*n*) of individuals, person‐years and number of fracture events. Fracture events incidence rate (IR) and incidence rate ratio (IRR) are presented with 95% confidence intervals (95% CI). We considered a statistically significant difference in IRRs between the intervention and control groups to exist when the 95% CI for the IRR did not include 1.00 (highlighted in bold text).

## 4 Discussion

This study indicates that 6–9 years of daily school PA is associated with a lower fracture risk than expected by age at the end of the intervention, but not after termination of the intervention.

Most studies evaluating the association between PA and fracture risk in childhood are observational [29, 30, 31, 32]. Self‐reported daily vigorous PA was in one of these studies linked to a high fracture risk [29], as was sport participation [30]. Other studies oppose the view when reporting no association between self‐reported level of PA and fracture risk in childhood [31]. In contrast, another study reported an association between low self‐reported PA and a high risk of fracture among girls [32]. In summary, data on the association between PA and fracture risk in childhood are contradictory.

The POP study is, to our knowledge, the only prospective controlled PA intervention study that uses not only surrogate endpoints such as bone mass and muscle strength, but actual fracture incidence as an endpoint variable [21, 25]. We have previously reported that 6–9 years of daily PA during the compulsory school years (ages 7–15) is associated with benefits in both bone mass and muscle strength [20], and that the bone mass benefits remained at the last evaluation, a mean of 7 years after termination of the program [22]. This is of great interest as high bone mass is associated with low fracture risk in both children [10] and adults [11, 33]. An earlier report from the POP study suggested that initiating daily PA in school may be associated with a temporary increase in fracture incidence, followed by a gradual reduction in fracture risk during the intervention [21]. However, the study evaluated a smaller sample than the current study, did not assess the same individuals across the first nine school years, did not account for official national mortality and migration data, and did not evaluate fracture risk after termination of the PA intervention. These weaknesses were addressed in the current study, in which we found that it appears possible to implement daily PA in school at a mean age of 7 without temporarily increasing fracture incidence. Furthermore, the current study also indicates that the previously reported lower fracture risk among children in the intervention compared to children in the control group not only exists in the 8th grade [21], but actually during the entire lower secondary school period (grades 7–9). The current study further indicates that the reduced fracture incidence in lower secondary school does not remain after daily PA is terminated. That is, the previously reported residual benefits in bone mass with daily school PA for a mean of 7 years after the PA program was terminated [22], are not reflected by a low relative fracture incidence. We speculate that, since data from the POP study indicate that individuals in the former intervention group continue with a higher duration of PA after the compulsory school years, compared to individuals in the control group [34], they may also be exposed to more trauma through sports activities. The view is supported by reports showing that active athletes (during a period when being exposed to sports related trauma) have higher bone mass [35, 36] and higher fracture risk than controls [37], but after the active sport career still have a higher bone mass [35] but now lower fracture risk than controls [23]. The POP cohort should therefore be followed into older ages when low‐energy related fragility fractures exponentially rise [38], before we can determine if increased PA during growth may be one strategy to reduce the risk of osteoporosis and fragility fractures.

The study also provides some indications that daily school PA might affect fracture risk differently in boys and girls (Figure 1 and Appendix 1). However, the study was not powered for subgroup analyses. Further studies with larger samples may be needed to clarify the issue.

Study strengths include the prospective controlled study design, the population‐based inclusion, the possibility to screen the radiographic archives for all of those included at baseline, a long follow‐up period spanning both the intervention and post‐intervention period, and the availability of national statistical data making it possible to address migration and mortality. Other study strengths include the inclusion of only objectively verified fractures, thereby avoiding recall bias, and that fracture classification was done by the same observer in both the intervention and control groups across all included years. Further strengths, compared with the previous POP fracture study [21], include a larger sample size with more person‐years and evaluation of the same individuals throughout the entire intervention period.

Limitations include the lack of individual randomization. However, the schools refused this at study start, as it was deemed practically impossible during a period of 9 years. Another weakness is that not all children had 9 years of daily PA. The decision to reduce the school curriculum PA in lower secondary school from fall 2010 was taken by the school based on economic reasons. It would also have been an advantage to have data on organized and non‐organized PA outside school including during vacations, as well as when and where the fractures were sustained. This would have given us the opportunity to evaluate the proportion of fractures that occurred during school PA classes, thus addressing the safety concern more thoroughly. A further limitation is that fractures occurring during visits outside our region, with no follow‐up radiographs in our region, were missed. However, we have no indication that the proportion of missing fractures would differ between the groups. A larger sample size would have allowed us, with adequate power, to evaluate boys and girls separately. Registration of confounders such as body mass index, maturation status at different ages, dietary intake, medication, diseases, calcium intake and vitamin D levels [19] as well as the amount of trauma exposure for each individual would also have been beneficial.

In summary, this study indicates that daily school PA initiated at age 7 is associated with a lower relative fracture risk at the end of the intervention (ages 13–15) but not after the intervention is terminated. Our data indicate that daily school PA may possibly be one method to decrease fracture risk during peak fracture incidence at growth. The cohort should be followed for a longer period to determine whether daily school PA is associated with a reduction of relative fracture risk later in adulthood.

## 5 Perspective

Fractures represent a substantial public health burden, and their incidence is projected to increase with rising life expectancy. Identifying effective and affordable prevention methods without adverse effects is therefore a priority in public health. A school‐based PA program offers a practical way to reach large populations of children. Increasing PA during growth has been shown to enhance bone mass and muscle strength, with benefits persisting after the intervention ended [22]. However, reports on the association between PA and fracture risk are conflicting [29, 30, 31].

This study indicates that daily school PA compared to school PA 1–2 times per week from a mean age of 7 is associated with a reduced relative fracture risk during peak fracture incidence at growth. The study also raises the question if the reduction in relative fracture risk would persist into late adolescence and early adulthood with continued daily PA, a research question worth testing in future studies.

## Author Contributions

Jakob Rempe and Magnus K. Karlsson: Conceptualisation, data curation, funding acquisition, investigation, methodology, supervision, validation, and writing and review‐editing. Magnus Dencker and Otto Nilsson Wimar: investigation, data curation, methodology, and writing and review‐editing. Björn E. Rosengren: conceptualisation, data curation, formal analysis, investigation, methodology, and writing and review‐editing. Lars Jehpsson: conceptualisation, data curation, formal analysis, investigation, methodology, validation, and writing and review‐editing. All authors contributed to the article and approved the submitted version.

## Funding

Funding was received from FoUU (2025/2026) and Stig & Ragna Gorthons Foundation (2025/2026).

## Ethics Statement

The study was approved by the Ethics Committee of Lund University (D.nr. LU 453–98 and D.nr. LU 2015/118) and the Swedish Ethical Review Authority (D.nr. 2019–02965).

## Consent

The participants were informed about the study through daily newspapers and were instructed to contact our research laboratory if they did not wish to participate.

## Conflicts of Interest

The authors declare no conflicts of interest.

## Data Availability

Data and materials were registered confidentially and in accordance with Swedish and EU data protection rules. This study is based on sensitive individual‐level data protected by the Swedish Personal Data Act. Access to the full data is available upon request from the corresponding author, provided that the person interested in using it receives ethical vetting. However, some aggregated tables can be provided by the corresponding author upon request.

## Appendix 1

Sex‐specific incidence rate ratios (IRR) for fracture events when comparing incidence rate (IR) in the intervention group with IR in the control group during the intervention period (grades 1–9; ages 7–15, mean 6.9 years of daily school‐based physical activity), and in the post‐intervention period (ages 16–30). The blue and red points represent the point estimates of IRRs for fracture events, and the bars the 95% confidence intervals (95% CI). The gray area illustrates the intervention period, and the white area the post‐intervention period. We regarded the result as statistically significant if the 95% CI for IRR did not include 1.0.
